# HIV Focused Sexual Risk-Reduction Interventions Targeting Adolescent Boys and Young Men in Sub-Saharan Africa: A Scoping Review

**DOI:** 10.1007/s10461-023-04054-8

**Published:** 2023-07-05

**Authors:** Roselyn Kanyemba, Kaymarlin Govender, Armstrong Dzomba, Tivani P. Mashamba, Joanne E. Mantell

**Affiliations:** 1 Health Economics and AIDS Research Division (HEARD), University of KwaZulu Natal, Durban, South Africa; 2 MRC/Wits Rural Public Health and Health Transitions Research Unit (Agincourt), School of Public Health, Faculty of Health Sciences, University of the Witwatersrand, Johannesburg, South Africa; 3 Faculty of Health Sciences, University of Pretoria, Pretoria, South Africa; 4 HIV Center for Clinical and Behavioral Studies, New York State Psychiatric Institute and Department of Psychiatry, Columbia University Irving Medical Center, New York, USA; 5 Present Address: School of Social Sciences, University of KwaZulu Natal, Durban, South Africa

**Keywords:** Sexual risk behaviours, Interventions, HIV prevention, Adolescent boys and young men, Sub-Saharan Africa

## Abstract

Adolescent girls and young women’s exceptionalism with HIV interventions has left adolescent boys and young men (ABYM) trailing behind, thus becoming a marginalized and underserved population. The scoping review aimed to provide an overview of interventions that have targeted sexual risk behaviors in ABYM in Sub-Saharan Africa (SSA) over the previous 21 years with critical insights on ‘what works’ in preventing the sexual transmission of HIV. A scoping review guided by Arksey and O’Malley’s (in Int J Soc Res Methodol 8(1):19–32, 16) framework and the 2015 Johanna Briggs Institute’s guidelines was conducted. A search of literature published between 2000 and 2020 was reviewed and twenty nine interventions from nine Sub Saharan African countries that met the eligibility criteria were reviewed. The review provides evidence on the successes and the limitations of sexual risk behavior interventions for ABYM in SSA. There is clear and consistent evidence that interventions reduce sexual risk behaviors in adolescent boys and young men. Their efficiency seems to grow with the length and intensity of the intervention. Positive effects were generally observed in condom use and on measures of HIV knowledge, attitudes and sexual behaviors as well as uptake of HIV tests and voluntary male circumcision. This review shows that sexual-risk interventions engaging men and boys in SSA are promising and warrant further rigorous development in terms of conceptualization, design and evaluation.

## Introduction

Adolescent boys experience increased HIV prevalence as they age, and adolescence may be a critical time for the formation of attitudes toward sex and sexual behaviors [1]. In sub-Saharan Africa (SAA), the epicenter of the HIV epidemic, 43% of boys and 53% of girls have had sex before age 18 in the period between years 2000 and 2015 [2]. Despite adolescent boys and men (ABYM) being at lower risk of HIV acquisition than girls, they are still at risk due to a combination of high frequency of sexual behaviors including condomless sex, sex under the influence of alcohol, and multiple sexual partnerships as well as poor health-seeking behaviors, such as not getting treatment for sexually transmitted infections (STI) and not testing for HIV [3]. There were 46% fewer deaths due to AIDS-related illness among girls and women in 2019 than in 2010, compared to 32% fewer deaths among ABYM over the same period, indicating a worse AIDS mortality risk for ABYM than for AGYW [4]. Indeed, adolescents experience complex physical, psychological, and social changes during the transition from childhood to adulthood. They encounter legal constraints (e.g., legal age of consent to HIV testing and counseling ranges between 12 and 18 years in SSA), cultural inhibitions against sexuality education, and material poverty-inducing exploitative sexual relationships. These factors restrict adolescents’ access to HIV services at a time when they are becoming sexually aware and lack knowledge and skills, thus enhancing their risk of HIV and other STI. The vulnerability to HIV and mortality among ABYM in SSA is exacerbated by their low social and economic position, as Gafos points out [5]. Their developmental, social and economic challenges highlight the urgent need to document and assess sexual risk-reduction interventions in the published literature targeting ABYM aged 10–24 years.

ABYM are seldom addressed in HIV prevention policies and programs. Several reviews have assessed the impact of HIV on adolescents in general, but few have focused on ABYM [6, 7]. Much of the available data do not disaggregate adolescent boys from adult males or adolescents in general; yet the contexts and life challenges of ABYM can be markedly different from those of other ages or genders [8–10]. Therefore, a scoping review is warranted to assess interventions targeting ABYM in SSA. Scoping reviews are a “preliminary assessment of potential size and scope of available research literature” [11, p. 95]. They aim to identify the nature and extent of research evidence and identify gaps in the literature so as to inform policy and practice.

This review complements other published reviews of sexual risk behaviors among adolescents and young people in SSA [12, 13] but differs from previous reviews in three major ways. First, the current review focuses on interventions that either target ABYM exclusively or includes the ABYM population and disaggregates results by age and gender. This is appropriate given the high emphasis placed on AGYW-focused HIV prevention interventions (e.g., DREAMS interventions being implemented in 10 SSA countries) [14] and that previous studies have combined ABYM data with AGYW or older male data [9, 15]. Second, the scope of the review is extended to include biomedical, behavioral and structural interventions that mitigate HIV risk for ABYM since previous reviews have tended to focus on psychosocial behavior change interventions only, for example [12]. Third, unlike previous reviews which have focused on either out-of-school or in-school interventions, the current review includes both in- and out-of-school data. Findings based on this review have implications for amplifying and fine-tuning the delivery of differentiated HIV prevention strategies for ABYM.

More specifically, the aim of this review is to provide an overview of interventions that have targeted sexual risk behaviors of ABYM in SSA between 2000 and 2020, with critical insights on ‘what works’ in preventing risky sexual behavior that could lead to HIV transmission. The central research questions of the review are as follows: What evidence-based HIV-directed sexual risk-reduction interventions exist for ABYM in SSA over the review period in the published or grey literature, and how effective have these interventions been in mitigating HIV risk?

## Methods

The methodological framework proposed by Arksey and O’Malley was adopted to guide the scoping review [16]. This framework emphasizes (a) identifying the research question, (b) identifying relevant studies, (c) selecting eligible studies, (d) charting of data, and (e) collating, summarizing and reporting the results, and supports the comprehensive ‘mapping’ of relevant literature in the field of interest. The study used the population, concept and context framework recommended by the Joanna Briggs Institute for Scoping Reviews [17].

### Inclusion Criteria

#### Characteristics of Study Population

In this review, we only considered ABYM between the ages of 10 and 24 years. We focused solely on ABYM in response to the lesser focus on HIV interventions targeting ABYM [7]. Since 2015, HIV interventions in SSA have mainly centered on AGYW. In this review, studies included sub-populations within a broad age range: males in early (10–14 years), middle (15–17 years) and late adolescence to young adulthood (18–24 years). We included studies that comprised AGYW only if results were disaggregated by gender and age.

#### Studies of Interest

We included studies evaluating HIV behavioral, biomedical, social and structural sexual risk-reduction interventions, detailing their effectiveness in reducing sexual risk among ABYM 10–24 years. The comparators could be different interventions or strategies: (a) inter- and intra-men groups; (b) within or between interventions (behavioral, biomedical, or structural); or (c) across gender, such as outcomes for ABYM vs. AGYW.

#### Context

The context of this review encompasses both location and time dimensions. Studies from SSA (i.e., a constituent of the World Health Organization Africa region most severely affected by HIV) between 2000 and 2020 were considered for the review.

#### Types of Studies

In this review, we included peer-reviewed and published data that were based on qualitative, quantitative and mixed methods research. Experimental- and quasi-experimental studies, randomized controlled trials, evaluation surveys, controlled before and after studies, and impact evaluations were included. Only English-language studies were considered due to a lack of resources to analyze studies published in other languages.

### Search Strategy

To execute the search for relevant studies, we followed three main steps recommended by the Joanna Briggs Institute [17]. The first step was a preliminary search of relevant databases, namely Cochrane Database of Systematic Reviews, Google Scholar, Joanna Briggs Library, MEDLINE/PubMed, Scopus, International Initiative for Impact Evaluation (3ie), and Web of Science. This was followed by an analysis of text words contained in the title and abstract, and of the index terms used to describe the article. We then conducted a second search using all identified keywords and index terms across all included databases. Finally, the reference lists of all identified reports and articles were searched for additional studies.

### Study Selection

The protocol and review team comprised two screeners and two reviewers. The first level of screening targeted only the title and abstract of citations. Both screeners conducted a comprehensive title screening by searching and uploading all literature search results on Endnote 20 software. These were reviewed by both reviewers; conflicts, duplications, and discrepancies were resolved. The literature was grouped into categories (Category 1—Relevant, Category 2—Not relevant, and Category 3—Potentially relevant). Abstracts were grouped under Category 3 if age and gender were not specified. The full-text articles of studies that satisfied the inclusion criteria (Category 1) were obtained. A final decision on inclusion was made by both reviewers and any uncertainties were resolved. All citations deemed relevant after the title and abstract screening were obtained for subsequent review of the full-text article. For articles that were not fully available electronically (abstracts only), the corresponding authors or journals were contacted for assistance in procuring the article. All articles deleted from the Endnote library were saved in a separate folder to ensure the reproducibility of the study. Table 1 presents the search string used to identify relevant studies.

**Table 1:** Keywords and search strategy

Adolescent terms	Adolescen* **OR** minor* **OR** juvenile* **OR** young adult* **OR** young man* **OR** boy* or teenager* Adolescent **OR** Adolescent health. Sexual* **OR** Risk Behaviors* intercourse **OR** Sex Education for ABYM
Topical keywords	HIV*education **OR** prevention* **OR** infection* programmes/strategies for ABYM **OR** youth; Sexually transmitted diseases/**OR** Reproductive Health/**OR** sex education **OR** adolescent sexual health services OR HIV infections OR condoms **Or** Circumcision OR reproductive behavior **OR** unsafe sex **OR** coitus OR reproductive behavior
Outcome measures	Search terms as: knowledge, attitude, intentions, behavior, self-efficacy designed to identify program trials measuring biological, behavioral, cognitive, attitudinal or other outcomes, not just HIV incidence, will be used in the search process

## Results

**Fig. 1:**
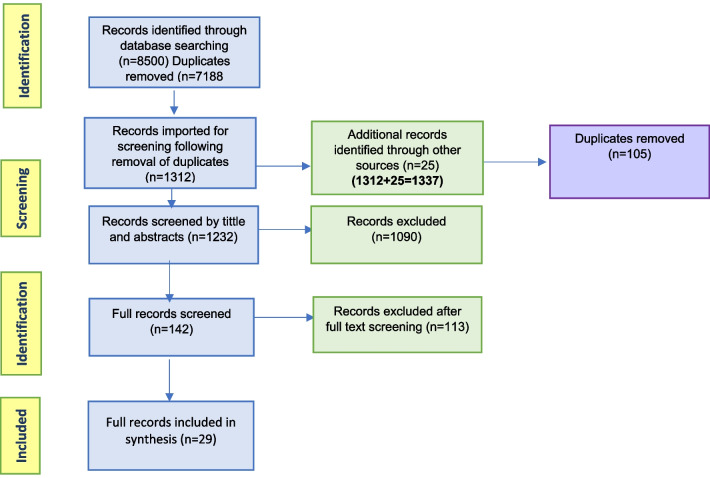
Flow chart for result of search and study selection

The search strategy identified 8500 records through database searching. Duplicates were removed and 1312 citations were imported for screening. An additional 25 records were identified through other sources. These citations were screened according to the title and 105 duplicates were removed. The resultant 1232 were further screened based on abstract relevance, with 1090 records being excluded. The remaining 142 abstracts were retrieved as full records and further subjected to screening according to the inclusion criteria; 113 records were excluded, yielding 29 records for inclusion in the review. Figure 1 presents a flow chart of search and study selection results.

### Quality Assessment of Eligible Studies

The study quality was assessed independently by two reviewers (RK and AD) using the methodological quality criteria Mixed Methods Appraisal Tool (MMAT) as outlined in Souto et al. [18] and Hong et al. [19]. Table 2 shows the assessment of the 29 studies included in the review. Overall, the quality of included studies was excellent, with 18 of 29 studies scoring 80–100%, six studies scoring 60% (i.e., fair to intermediate), and the last five being of poor quality, scoring 40%. Similarly, risk of bias was assessed across several domains as presented in Table 3, indicating studies have low to intermediate concerns overall.

**Table 2:** MMAT study quality assessment

Study	S1	S2	1.1	1.2	1.3	1.4	1.5	2.1	2.2	2.3	2.4	2.5	5.1	5.2	5.3	5.4	5.5	Rating (%)
Myers et al. [20]	y	y	y	n	y	y	y											80
Collinge et al. [21]	y	y	y	n	y	y	y											80
Pettifor et al. [22]	y	y						y	y	y	n	y						80
Figueroa and Kincaid [23]	y	y											n	n	y	y	n	40
Jewkes et al. [24]	y	y						y	y	y	c/t	y						80
Dithlopo et al. [25]	y	y											y	n	y	y	n	60
York [26]	y	y	y	y	y	y	y											100
Kajubi et al. [27]	y	y											y	y	y	y	n	100
Agha and Vanrossem [28]	y	y											y	y	y	y	y	100
Pulerwitz et al. [29]	y	y											y	y	y	y	n	80
Karim et al. [30]	y	y											y	y	y	y	n	80
PATH [31]	y	y											y	y	n	y	n	60
Dancy et al. [32]	y	y											y	y	y	y	n	80
Stern et al. [33]	y	y											y	y	y	y	n	80
Coffman et al. [34]	y	y						n	y	y	c/t	y						60
Mmbaga et al. [35]	y	y						y	y	y	y	y						100
Weiss et al. [36]	y	y						y	y	y	c/t	n						60
Rotherman-Borus et al. [37]	y	y						y	y	y	c/t	y						80
Kaufman et al. (a) [38]	y	y						y	y	n	n	n						40
Gibbs et al. [39]	y	y						y	y	y	n	y						80
Botcheva et al. [40]	y	y						y	y	y	c/t	y						80
Kyegombe et al. [41]	y	y						y	n	y	c/t	n						40
Jemmott et al. [42]	y	y						y	y	y	y	y						100
Hallman et al. [43]	y	y											y	n	n	n	y	40
Shanaube et al. [44]	y	y						y	y	y	c/t	n						60
Aderibigbe and Araoye [45]	y	y											y	y	y	y	y	100
Burnett et al. [46]	y	y						n	c/t	n	y	y						40
Kaufman et al. (b) [47]	y	y											y	c/t	y	y	y	80
Fritz et al. [48]	y	y											y	y	n	n	y	60

For complete guide on MMAT study abbreviations and number representative please refer to MMAT study guide explained in Reference [18]

**Table 3:**
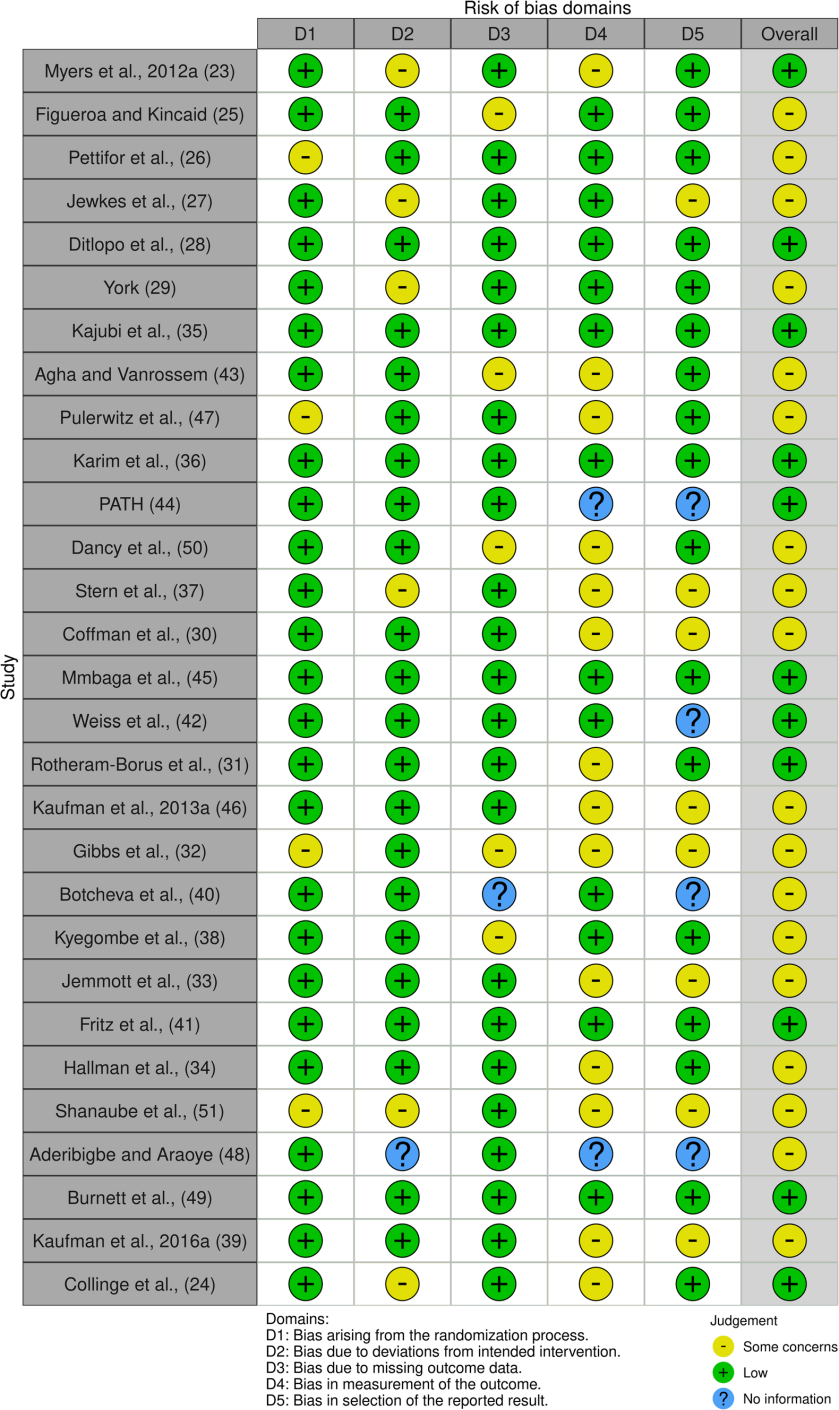
Risk of bias domains

### Data Collection

We manually extracted the data on intervention design, sample size, length of follow-up, and sexual-risk related outcomes for all 29 studies using data retrieval rubrics from the Joanna Briggs Institute of Meta-Analysis of Statistics Assessment and Review Instrument Quantitative (JBI-MAStARI) for quantitative studies [49] and Briggs Institute Qualitative Assessment and Review Instrument (JBI-QARI) for qualitative studies [50, 51].

### Findings

In total, 29 studies were eligible for inclusion in this review. An overview of the sexual risk behavior interventions targeting ABYM in SSA and specific details about populations, study designs and key outcomes related to the study aim and research questions are displayed in Tables 4, 5, and 6.

**Table 4:** Description of the interventions in the scoping review

References	Intervention name, duration, and country	Setting and evaluation design	Target population
Myers et al. [20]	*Brothers for Life (B4L)* Duration: 2 years (August 2009–August 2011) Country: South Africa	Context: Urban, rural, and peri-urban areas of Cape Town Design: Experimental design	ABYM aged 15–24 and older men aged 30+
Collinge et al. [21]	*Game of Life Campaign (sub-campaign of Brothers for Life Campaign* Duration: 12 months 2010–2011 Country: South Africa	Context: Urban, rural, and peri-urban areas of Cape Town Design: Experimental design	Males aged 15–49 years
Figueroa and Kincaid [23]	*VMMC (sub-campaign of B4L)* Duration: 3 years 2009–2012 Country: South Africa	Context: Urban, rural, and peri-urban areas Design: Exploratory design	ABYM aged 15–24 years
Pettifor et al. [22]	*One Man Can Campaign* Duration: 25 months May 2012–June 2014 Country: South Africa	Context: Rural Gender-Transformative Intervention (community) Design: Cluster randomized controlled	Males aged 18–35 years
Jewkes et al. [24]	*Stepping-Stones* Duration: 12 months March 2003–March 2004 Country: South Africa	Context: Rural Eastern Cape, South Africa Design: Randomized controlled trial	ABYM and AGYW (mostly in-school) aged 16–23 years
Ditlopo et al. [25]	*Men as Partners Programme* Duration: 12 months 2012 Country: South Africa	Context: 16 townships in urban Soweto Design: Evaluation survey	Heterosexual males aged 15–34 years
York [26]	*Khanyisa Programme* Duration: 12 months 2011–2012 Country: South Africa	Context: 1 community in rural KwaZulu-Natal Design: Exploratory design	ABYM aged 15–24 years
Kajubi et al. [27]	*Condom Technical Skills Education* Duration: 12 months 2001–2002 Country: Uganda	Context: Peri-urban (2 communities near Kampala) Design: Quasi-experimental controlled trial	Men aged 18–30 years
Agha and Van Rossem [28]	*The Zambia Peer Sexual Health Intervention* Duration: 9 months 2000–2001 Country: Zambia	Context: Lusaka Urban secondary schools (3 intervention and 2 control) Design: Quasi-experimental	ABYM and AGYW aged 14–23 years
Pulerwitz et al. [29]	*PEPFAR Male Norms Initiative* (*MNI*) Duration: 6 months June–November 2008 Country: Ethiopia	Context: 3 low-income sub-cities of Addis Ababa (urban) Design: Quasi-experimental	ABYM aged 15–24 years (not married, highly educated)
Karim et al. [30]	*African Youth Alliance Programme* (*AYA*) Duration: 5 years 2000–2005 Country: Uganda	Context: Peri-urban district Design: Static-group comparison design	ABYM and AGYW aged 17–24 years
PATH [31]	*PATH-Kenyan Scouts* Duration: 12 months March 2010–March 2011 Country: Kenya	Context: 115 urban and rural schools Design: Quasi-experimental design	ABYM and AGYW ‘scouts’ in Kenya aged 15–18 years
Dancy et al. [32]	*Mzake ndi Mzake Kuunikira Achinyamata* (*MMKA*) (*Friend to friend guiding the youth)* Duration: 2 years 2005–2007 Country: Malawi	Context: Rural Malawi Design: Quasi-experimental design	ABYM and AGYW aged 13–19 years
Stern et al. [33]	*Learning Centre Initiative Reproductive Health* Duration: 3 years 2011–2013 Country: Uganda	Context: Urban Design: Exploratory design	Heterosexual men aged 18–54 years
Coffman et al. [34]	*Healthwise Programm*e Duration: 1 year 2001–2002 Country: South Africa	Context: Urban school-based intervention in 4 schools Design: Randomized controlled trial	ABYM and AGYW aged 13–18 years
Mmbaga et al. [35]	*PREPARE Intervention* Duration: 3 years 2011–2014 Country: Tanzania	Context: Urban school-based intervention, 13 schools Design: Cluster randomized controlled trial	ABYM aged 12–14 years
Weiss et al. [36]	*The Spear and Shield Project* Duration: 3 years 2012–2014 Country: Zambia	Context: Urban in 13 community health centers Design: Cluster randomized trial	Males aged 18 years and older
Rotheram-Borus et al. [37]	*Champions League* Duration: 6 months April–September 2012 Country: South Africa	Context: Urban soccer-based intervention in 2 Cape Town neighborhoods Design: Randomized controlled trial	Unemployed ABYM aged 18–25 years
Kaufman et al. (a) [38]	*Make the Cut* + Duration: 8 months March–October 2014 Country: Zimbabwe	Context: Peri-urban soccer-based Design: Randomized controlled trial	Secondary school ABYM aged 14–20 years
Gibbs et al. [39]	*The Stepping-Stones and Creating Futures intervention* Country: South Africa Duration: 12 months September 2015–September 2016	Context: Urban informal *settlement* Design: Cluster randomized controlled trial	ABYM and AGYW out-of-school and unemployed, aged 18–30 years
Botcheva et al. [40]	*Grassroots HIV Intervention Programme* Duration: 5 months in 2003 Country: Zimbabwe	Context: Urban in-school program Design: Cluster randomized controlled trial	ABYM aged 13–18 years
Kyegombe et al. [41]	*SASA!* Duration: 5 years 2007–2012 Country: Uganda	Context: Urban phased community mobilization in 8 communities Design: Cluster randomized controlled trial	Males and females aged 18–49 years with equal number of males and females
Jemmott et al. [42]	*Men, Together Making a Difference!* Duration: 3 years 2007–2010 Country: South Africa	Context: 206 neighborhoods in Cape Town Design: Cluster randomized controlled trial	Men aged 18–45 years
Fritz et al. [48]	*Sahwira HIV Intervention Programme* Duration: 5 years 2002–2007 Country: Zimbabwe	Context: 24 urban communities Design: Randomized controlled trial	Men 18 years and above visiting beer halls
Hallman et al. [43]	*Siyakha Nentsha Programme* Duration: 18 months spanning 2008–2009 Country: South Africa	Context: Peri-urban Design: Quasi-experimental design	School-age ABYM and AGYW aged 14–16 years
Shanaube et al. [44]	*(PopART) for Youth Study* Duration: 12 months 2015–2016 Countries: Zambia and South Africa	Context- Urban Design: Community randomized trial	ABYM and AGYW aged 10–19 years
Aderibigbe and Araoye [45]	*No name health education program* Duration: 6 months January–June 2006 Country: Nigeria	Context: 6 urban public secondary schools Design: Quasi-experimental design	ABYM and AGYW aged 10–19 years
Burnett et al. [46]	*It’s Our Future Too* Duration: 2 years 2007–2009 Country: Swaziland	Context: High school-based curriculum Design: Randomized controlled trial	ABYM and AGYW aged 15–17 years
Kaufman et al. (b) [47]	*Fataki Campaign* Duration: 2008–2011 (3 years) Country: Tanzania	Context: 21 urban and rural regions in Tanzania mainland Design: Explanatory design	ABYM and AGYW aged 15 years and older

References	Aims/objectives	Intervention description
Myers et al. [20]	To encourage and reinforce positive behaviors through promoting alternative definitions of masculinity. Dominant masculinities prevailing exposed men to HIV risk and manifested through violent behaviors toward women and male sexual entitlement	Multifaceted community mobilization campaign to promote positive shifts in existing gender roles and to encourage men to help each other in being responsible partners and taking action to prevent HIV. This involved mass media campaigns, advocacy, partnership-building and community radio talk-shows. Gender-based violence adverts and distributions of VMMC information leaflets complemented these efforts
Collinge et al. [21]	Promote messages about sexual health and non-risky behavior	Using prominent sports personalities to drive a mass media and community mobilization campaign on adopting safe sexual behavior. This was delivered via numerous TV and radio advertisements; Multiple short adverts featuring sport stars’ print media visibility through billboards, on major transit routes, at airports and near major stadiums. The community mobilization component involved condom distribution at 2010 World Soccer World Cup fan parks
Figueroa and Kincaid [23]	Raise awareness about benefits of MMC and generate demand	A multi-faceted intervention based on community mobilization approach to promote uptake of medical male circumcision. The campaign involved and equipped local organizations to promote this HIV risk-reduction practice in the homes, clinics, religious congregations, workplaces, taxi ranks and sports clubs in participants’ communities. B4L worked with individuals and with social networks to convey safer SRH practices
Pettifor et al. [22]	Change negative gender norms and HIV risk and increase awareness about the relationship between gender inequities and HIV and encourage the community, and especially men, to take action to address negative gender norms and HIV risk	Community education and mobilization campaign implemented through door-to-door home visits, street soccer and soccer tournaments, acting ambush theatre (i.e., actors act out a scene in a public place, then reveal their cover and engage the crowd in conversation over the scene they witnessed) and painting murals in communities, workshops, and leadership engagement meetings, soccer matches
Jewkes et al. [24]	Improve sexual health by using participatory learning approaches to build knowledge, risk awareness, and communication skills as well as to stimulate critical reflection	A series of educational workshops, involving critical reflection, role-play, and drama drawing the everyday reality of participants’ lives and a final community meeting. The program spanned about 50 h and ran for 6–8 weeks
Ditlopo et al. [25]	Transform the behaviors of men and the norms of masculinity which impact violence and risk behavior toward sex partners	Community mobilization, education, and advocacy campaign. Participants attended workshops over a 9-month period and exposed to community mobilization content amounting to at least 35 h
York [26]	To address masculinity, inequality, gender, violence, and HIV/AIDS among Zulu youth, exploring mechanisms to transform toxic male masculinities in a culturally appropriate manner	Gender-transformative program education through a series of experiential learning exercises which covered trust-building, gender and social/cultural expectations, HIV and gender, gender and power, violence, attitudes about rape, tests of courage and the modelling of new kinds of courage, relationship control, intimate partner violence and coping, sexual violence perpetrated by sexual abuse, knowledge about HIV/AIDS, STI and access to HIV testing, participation in care and support and prevention of HIV
Kajubi et al. [27]	Improve accessibility plus condom-use skills, HIV prevention	A condom promotion program instilling condom technical use skills in workshops and promoting condom use
Agha and Van Rossem [28]	Reduce risky sexual behavior and sexual transmission of HIV among adolescents	Peer education sessions about HIV through poems and group discussions
Pulerwitz et al. [29]	Promote gender-equitable norms and reductions in intimate partner violence (IPV)	Interactive group education and community mobilization program adapted from the Men as Partner (MAP) program. This involved engagement activities to raise awareness and promote community dialogue through distributing leaflets and newsletters, music and drama skits, condom distribution and monthly meetings. Other activities included role-play practice and guided discussions on gender norms and HIV risk
Karim et al. [30]	Increase the use of high-quality SRH services by establishing youth-friendly health facilities, extending outreach services, establishing peer providers of services, and institutionalizing an appropriate SRH service curriculum in the Ministry of Health in-service training	Behavior-change communication, providing youth-friendly clinical services, and coordinating policy and advocacy. Activities included mass media campaigns and engaging young people, community members, and stakeholders through networking activities, workshops, and student essay competitions and debates; interpersonal communication; folk and mass media, including drama; life planning skills programs for youth; peer education and counselling; and social marketing campaigns through community discussions
PATH [31]	Break gender barriers: Change gender norms of boys and men in Kenya	Merit-based program promoting equitable gender norms. Activities included outreach and camping trip where scouts (boys and girls) had to complete all the activity pack sessions and earn at least one other proficiency badge for practicing an activity traditionally carried out by the other gender
Dancy et al. [32]	Enhance HIV knowledge, attitudes, self-efficacy, and HIV risk-reduction behaviors	Group-based SRH education campaign to enhance HIV knowledge, attitude about HIV, self-efficacy for condom use and safer sex, and promoting intention to adopt HIV risk-reduction behaviors
Stern et al. [33]	Engage men in sexual and reproductive health as clients, equal partners, and advocates of change to increase men’s access	Knowledge-based intervention. Information dissemination campaign offering technical support for male sensitive SRH delivered via mobile phone messaging and on-site visits. Other dynamic locations and platforms to share information included churches, football tournaments, community outreach, drama, and distribution of posters
Coffman et al. [34]	Reduce sexual and alcohol risk behaviors	Educational curriculum incorporating specific lessons on attitudes, knowledge, and skills related to avoidance of substance abuse and sexual risk (e.g., ideal relationships, condom use, realities, and myths of drug use)
Mmbaga et al. [35]	Focus on gender and power, relationships, assertiveness and communication, decision-making, risk-taking, violence, self-protection and support reducing sexual risk behavior and IPV among adolescents; delaying sexual initiation and promoting condom use; and linking adolescents to information and services that may foster healthy sexuality, health information and services. Such services include access to condoms, contraception, STI management and HIV	In-school cluster activities included role-playing and drama workshops
Weiss et al. [36]	Medical male circumcision	Guided group sessions promoting VMMC as a perpetual risk-reduction method, including reduction of high-risk sexual behavior under the influence of alcohol or drugs
Rotheram-Borus et al. [37]	Engage men in HIV testing and reducing substance use through soccer, role play	Interactive soccer sessions promoting healthy lifestyles through a ‘no drug abuse’ mantra. This was accompanied by random alcohol and drug tests at the beginning of soccer matches
Kaufman et al. (a) [38]	Promote VMMC to facilitate linkage to male circumcision	Interactive soccer-themed educational session building on the popularity of soccer among Zimbabwean males to initiate discussions motivating males to take up VMMC
Gibbs et al. [39]	Transform gender attitudes and strengthen livelihoods	Participatory gender-transformative and livelihoods strengthening intervention delivered over 21 sessions
Botcheva et al. [40]	Reduce the spread of HIV/AIDS by training adult soccer players to educate at-risk youth about HIV/AIDS	A 4-day educational intervention (with teaching sessions conducted twice weekly for 2 weeks); the educational intervention utilizes a classroom-based curriculum
Kyegombe et al. [41]	Prevent IPV and reduce HIV-related risk behaviors at the community level	Community mobilization intervention to engage communities to prevent IPV and promote gender equity
Jemmott et al. [42]	STI risk-reduction	Educational intervention employing interactive exercises, games, role-playing, take-home tasks and watching education content
Fritz et al. [48]	Promote idea that men can and should take responsibility for their friends’ well-being by assisting each other to avoid high-risk sexual encounters associated with drinking at the beer hall	One-on-one, small group interactions, and educational events driving the theme of men helping their male friends to avoid risk and test for HIV
Hallman et al. [43]	Building knowledge and skills that would empower girls and boys to work together, interact socially, learn to respect one another, and thus move beyond objectification of the opposite sex	Orientation program to improve life-long skills and well-being
Shanaube et al. [44]	Acceptability and uptake of a combination HIV prevention package (i.e., including condom use and VMMC) among young people	Intervention included the employment of youth counselors, training parents and clinic staff to strengthen HIV prevention school-based activities. These youth friendly HIV prevention activities functioned as hubs for educational materials and provision of and distribution of condoms, screening for STI’s, and referrals to voluntary medical male circumcision for HIV-negative men
Aderibigbe and Araoye [45]	Reduce risky sexual behaviors and improve condom use	Health education sessions consisting of lectures, film show and IEC materials. Topics focused on HIV/AIDS and sexual behaviors, including condom use and risk of exchange of sex for gifts
Burnett et al. [46]	Improve HIV-related knowledge, attitudes, and safer sexual behaviors, including HIV testing to change students’ behaviors to reduce HIV incidence and equip Swazi youth with skills that will enable them to attain employment and/or pursue a higher academic degree	Education and health four-pronged enrichment curricula: life skills for HIV awareness and prevention, computer technology, job readiness, community outreach. The program was held over a period of 13 half-day Saturday sessions, with 1-h per week for each of the four curricula
Kaufman et al. (b) [47]	Address cross-generational sex by mobilizing communities to intervene in cross-generational sex relationships	Mass media campaign launched on 15 radio stations—an average of 10–12 spots were aired at prime time each day, awareness banners placed in high traffic areas, posters hung near schools in 10 high prevalence regions

**Table 5:** Intervention outcomes (descriptive)

References	Factors associated with risk	Tools for measurement	Theory/model followed	Type of facilitators	Outcomes/impact post-intervention	Percent (%)	Mean
Myers et al. [20]	*Structural* e.g., Harmful male norms leading to GBV *Behavioral* e.g., Unprotected sex, inconsistent use of condoms, multiple partner sex, alcohol use *Biological* e.g., Lack of circumcision, HIV testing and treatment	*Self-reported* through dialogue in group discussions *Police register* drop in rape and violence reports *Clinic register* HCT, uptake of circumcision, condoms and accompanying women to antenatal clinic	*Theory* Positive Normativity and Social Learning Theory *Model* Social- Ecological Model, especially employing the effect of individual responses, influence of social networks, community resources and processes (notion of brotherhood)	1. Peer educators and facilitators 2. Brand ambassadors (e.g., celebrity stars)	*Impact-based*		
					1. Positive norm-based—a drop in propensity for domestic violence after a community dialogue 2. Increased requests for condoms 3. Reduction in concurrent multiple partnerships 4. People approaching Brothers for Life workers privately to seek help with partner abuse, excessive drinking or a sexual problem 5. Improved intention to get circumcised and use condoms		
Collinge et al. [21]	*Behavioral* e.g., Inconsistent condom use, alcohol consumption *Biological* e.g., Poor rates of HIV testing *Structural* e.g., Harmful gender norms leading to GBV	*Self-reported* through dialogue in group discussions *Police register* drop in rape and violence reports *Clinic register* HCT, uptake of circumcision, condoms and accompanying women to antenatal clinic *Biological* Rapid drug test	*Theory* Social Identity Theory for participants to identify with soccer celebrities and heroes and model behavior after their messages of behavior change	1. Sports celebrities from the 2010 World Cup 2. Trained soccer coaches who acted as role models	*Impact-based*		
					1. Widespread requests for condoms 2. Reduction in concurrent multiple partnerships (self-reported) 3. Drop in rape incidence 4. Reduction in alcohol and drug abuse		
					5. Condom use improved	↑55% vs. 49%	
Figueroa and Kincaid [23]	*Behavioral* Incorrect and inconsistent condom use, multiple sex partners *Biological* MMC, HTC *Structural* Lack of education on linkages between HIV/TB, knowledge of signs and symptoms of TB and other opportunistic infections	*Self-reported* through group dialogue	*Model* Biomedical	Trained facilitators and trained medical personnel	*Outcome-based behavioral*		
					1. Improved levels of knowledge of the protective effect of MMC against HIV infection increased More uncircumcised male participants were aware that circumcision reduced the risk of HIV infection compared to baseline	44%** vs. 8% in 2009	
					More men were aware that circumcision reduced the risk of other STIs	48%**	
					2. Overall increased intent to be circumcised in 12 months 3. Intention to get circumcised	33%**	
					4. Circumcised was highest in the 16–24-year age group where it increased	46%**	
Pettifor et al. [22]	*Structural* Negative gender norms fueling HIV risk, poor knowledge about the relationship between gender inequities and HIV 2. Improve self-efficacy to reduce HIV/AIDS 3. Encourage VMMC	Gender Equitable Men (GEM) Scale was used in the surveys to assess knowledge, attitudes, and behaviors related to gender norms	No theory/model mentioned	1. 2 supervisors 2. 15 community mobilizers (both sexes) 3. At least one community action team (CAT) in each community, which on average included about eight members 4. Intervention manager 5. Engaging community and religious leaders	*Outcome-based behavioral*		
					Levels of knowledge of the protective effect of MMC in terms of HIV infection increased		–
Ditlopo et al. [25]	*Structural and behavioral* Men’s gender attitudes, norms, and behaviors; aspects of gender dynamics in relationships; and increasing male involvement in GBV and HIV prevention and HIV care and support activities *Behavioral* Multiple partner sex, poor HCT	Self-reported through surveys, questionnaires and focus group discussions	*Model* Framed on the Human Rights Framework Approach	Peer educators	*Outcome-based* Several structural and behavioral factors were improved post- vs. pre-intervention:		
					1. Fewer men believed that men should make final household decisions	50%* vs. 38%	
					2. Fewer men believed that women who carry condoms on them are easy	36%* vs. 20%	
					3. Fewer men consistently used condoms in the last 6 months	19%**vs. 38%	
					4. 13% vs 40% used condoms during sex with non-regular partner	13%** vs. 40%	
					5. None believed that a person could transmit HIV by hand shaking	0%* vs. 5%	
					6. More men believed that having sex with healthy-looking partners reduces transmission risk	15%* vs. 6%	
					7. More men believed that a person infected with HIV by donating blood	52%* vs. 35%	
					8. Fewer men ever discussed with partner about having an HIV test	25%** vs. 59%	
York [26]	*Structural* Norm-based harmful norms contributing to inequality, gender violence and HIV/AIDS among Zulu youth	Self-reported through questionnaires and focus group discussions	*Theory* Transformative Learning Theory *Model* Concept of ubuntu	Trained facilitators	*Impact-based*		
					1. Decrease in GBV and sexual risk-taking behavior with no effect-size data		
Kajubi et al. [27]	*Structural* Poor SRH accessibility. Poor condom use, HIV and STI prevention	Condom acquisition as measured by coupon redemption	No model/theory mentioned	Nurses	*Outcome-based* Differences in outcomes for the intervention vs. control groups of men:		
					1. Uptake of condoms improved in the intervention group	89%*	
					2. Increase in number of sexual partners in the intervention group (mean difference) and decrease in the control		0.31 vs 0.17
					3. Larger decrease in number of sex partners among those in the control mean difference for the intervention group		0.02 vs 0.13
Pulerwitz et al. [29]	*Structural* Norm-based. harmful norms leading to intimate partner violence (IPV)	*Gender norms* Gender-Equitable Men (GEM) Scale *IPV* measured according to physical or sexual violence committed during the preceding 6 months and any type of violence (physical, sexual, or psychological) committed during the preceding 6 months	*Theory* Theory of Gender and Power	Trained facilitators	*Impact-based behavioral*		
					1. Percentage reporting physical or sexual violence toward their partner decreased	↓to 16%* from 36%	
					2. Percentage of young men reporting physical or sexual violence decreased after exposure to community engagement intervention only	↓to 18%* from 36%	
					3. Percentage reporting any type of violence decreased	↓to 37%* from 60%	
Karim et al. [30]	*Structural* Lack of youth-friendly health services *Behavioral* Poor SRH behavior	Self-reported exposure through questionnaire	*Model* Framed on the Health Belief Model	Trained facilitators, trained healthcare providers	*Impact-based* *Behavioral* No significant impact on partner reduction among males exposed vs. not exposed to program:		
					1. Delayed sexual initiation	76% vs. 79%	
					2. Had fewer sex partners	70% vs. 76%	
					*Structural*		
					3. Expanded scope and coverage of SRH for adolescence	33% vs. 35%	
PATH [31]	*Structural* Harmful gender norms	*Attitudes* related to gender norms were measured using the Gender Equitable Men (GEM) Scale *GBV* WHO Multi-country Study on Women’s Health and Domestic Violence against Women *Sexual behavior* Self reported	No model/theory mentioned	Senior scout leaders	*Impact-based* *Structural* Attitude change in self-rated gender norms at endline vs. baseline:		
					1. Percentage believing that only women should preserve their virginity until marriage decreased	↓from 34%* to 19%	
					2. Belief that a woman should tolerate violence to keep her family decreased	↓from 51%* to 29%	
					*Behavioral*		
					3. Self-reported condom use increased	↑from 34%* to 47%	
Dancy et al. [32]	*Structural* Lack of HIV knowledge, harmful gender attitudes, poor self-efficacy *Behavioral* HIV risk- reduction behaviors	*General HIV knowledge* Index of 11 true/false dichotomous items score. HIV attitude—Hope scale. Self-reported on a researcher-developed scale	*Theory* Based on Social Cognitive Learning Theory and Theory of Reasoned Action	Trained facilitators	*Strength-based* *Structural*		
					Males had a significant increase in their knowledge about HIV and improvement on their self-efficacy for condom use		
Stern et al. [33]	*Behavioral* 1. Poor access and utilization of HIV counseling and testing 2. Poor utilization of voluntary medical male circumcision, reproductive health planning, testing and treatment of STIs, including HIV	Self-reported through surveys, questionnaires and focus group discussions	No theory/model mentioned	Peer educators and project staff	*Impact-based*		
					Demonstrable male involvement in sexual and reproductive health programs		
Coffman et al. [34]	*Behavioral* Alcohol and drug use, sexual risk behavior *Structural* Lack of knowledge on linkage between and alcohol drug abuse, harmful norms, sexual behavior, and HIV	Self-reported using a Likert scale	*Theory* Ecological Systems Theory	Trained teachers	*Outcome-based*		
					1. Boys compared to girls had improved knowledge about acquiring and using condoms		
Rotheram-Borus et al. [37]	*Biological* Lack of HIV testing and counseling *Behavioral* Drug and alcohol abuse *Structural* Poor self-efficacy due to lack of skills	*Drug abuse* Rapid drug testing. Alcohol use-AUDIT scale *Attitudes towards women* Respecting Women scale	No model/theory mentioned	Coaches were selected only if they were not regular attendees at their neighborhood shebeens or known alcohol or drug addicts	*Outcome-based* At the pre- vs. post-assessments:		
					Having 2+ sex partners did not decrease	40.8%** vs. 41.5%**	
					Substance abuse decreased	↓from 72.1% to 67.7%	
					HIV testing rates increased	↑from 19.1% to 26.7%	
Botcheva et al. [40]	*Structural* Lack of knowledge about HIV/AIDS, poor life-skills, masculinity norms, lack of knowledge of social support services	Self-reported through questionnaire	*Theory* Social Learning Theory	14 nationally known soccer players were trained as facilitators	*Strength-based* Improved student (both male and female) knowledge, attitudes, and perceptions of social support related to HIV/AIDS—pre-test vs. follow-up:		
					1. Condoms can help prevent you from getting AIDS	↑from 50 to 60%	
					2. You may get HIV/AIDS from donating blood	↑from 35 to 55%	
					3. I know what it means to have ‘protected’ sex	↑from 57 to 70%	
					4. I hug a person who has AIDS, I can be infected	↑ from 82 to 90%	
					5. I know where to go for the help for HIV/AIDS-related problems	↑from 46 to 75%	
					6. Listed 3 or 4 more people to talk about HIV/AIDS problems	↑from 33 to 62%	
Fritz et al. [48]	*Behavioral* Alcohol use, sexual behaviors, patterns of peer influence on sexual risk behavior, poor knowledge about HIV	Self-reported using FGDs	*Model* Grounded in the Zimbabwean cultural concept of the Sahwira, a particularly close and trusted friend	Volunteer peer educators	*Impact-based* No evidence that the intervention vs. control reduced other risks for HIV:		
					1. Mean number of partners		1.5 (1.3–1.7) v 1.5 (1.3–1.7)
					2. No differences in the number of condomless sex episodes with non-wife partners after intervention vs. controls (5.4 vs. 5.1 episodes)		5.4 vs 5.1
Hallman et al. [43]	*Structural* Financial instability, poor access to SRH	Self-reported through interviews	No theory/model mentioned	Young adult trained facilitators	*Impact-based*		
					1. Intervention boys were more likely to have remained sexually abstinent between survey rounds compared to control boys 2. Intervention boys who did have sex reported fewer sexual partners than did boys in the control group 3. Improved budgeting and planning skills in school-going boys and girls (14–16 years) only in the intervention group at 18-month follow-up		
Shanaube et al. [44]	*Structural* Lack of knowledge of HIV status, poor acceptability and uptake of HIV prevention and ART treatment in view of the 90-90-90 UNAIDS targets	Self-reported through questionnaire	No theory/model mentioned	Trained community health workers called community HIV care providers	*Outcome-based*		
					1. Increased % of boys knew their HIV status after intervention in Zambia and in South Africa, respectively	↑from 29.2% to 87.8% in Zambia ↑from 21.3% to 77.3% in SA	
					2. % on ART was greater for boys than girls in Zambia and in SA	9% vs. 4%	
					3. % coverage against the 1st 90 target	75–84% boys vs. 75–83% girls	
					4. % against the 2nd 90 target	73–86% boys vs. 68–86% girls	
Aderibigbe and Araoye [45]	*Behavioral* Risky sexual behaviors including transactional sex and inconsistent condom use	Self-reported through questionnaire	No theory/model mentioned	Not mentioned	*Outcome-based*		
					1. Slight increase in condom use among females vs. males post-intervention		

**p < 0.0001, *p < 0.05

**Table 6:** Intervention outcomes (Statistical results)

References	Factors associated with risk	Tools for measurement	Theory/model followed	Type of facilitators	Outcomes/impact post-intervention	Odds ratio (OR)	Relative risk (RR)	Beta coefficient (β)	Incidence rate (IR)
Jewkes et al. [24]	*Structural* Lack of knowledge and access to contraception and conception, negative gender norms, poor communication skills, improve linkage to care and ART *Behavioral* Risky sexual behavior, poor and inconsistent use of condoms	*Biological* Blood samples to determine HIV status. The Determine (Abbott Diagnostics, Johannesburg, South Africa) test was used as a screening test *Behavioral* Structured questionnaire administered by an interviewer in face‐to‐face interviews (self-reported) *Structural* Socio-economic status assessed using a scale capturing household goods ownership (TV, radio, and car), frequency of hunger, frequency of having meat *Childhood Trauma* modified childhood trauma questionnaire *Alcohol use* Alcohol Use Disorders Identification Test (AUDIT) scale	*Model* Participatory Learning Model	Project staff and same-sex trained facilitators trained nurses and counselors	*Outcome-based*				
					*Biological*				
					1. Stepping Stones did not reduce HIV incidence, but had an impact on several risk factors for HIV—notably, reducing HSV-2 incidence				(IR 0.67, 95% CI 0.46 to 0.97)*
					*Behavioral*				
					2. Lowered several reported risk behaviors in men e.g., number of sex partners	aOR − 0.0078, 95% CI − 0.0033 to 0.0001			
					3. Transactional sex with a casual partner	(aOR 0.39, 95% CI 0.17 to 0.92)*			
					4. > 1 incident intimate partner violence after 24-months	(aOR 0.62, 95% CI 0.38 to 1.01)			
					5. Drinking problem after 12 months	(aOR 0.68, 95% CI 0.49 to 0.94)*			
Agha and Van Rossem [28]	*Behavioral* Early sexual initiation, risky sexual behavior, e.g., multiple sex partners	Self-reported accounts through peer interaction	No model/concept mentioned	Trained peer educators	*Impact-based* Changes in knowledge and normative beliefs about abstinence and condom use observed after the intervention and sustained over 6-month period vs. baseline:				
					1. Appropriate for a woman to propose abstinence; increase in odds at 1st follow-up vs. baseline	aOR 1.97, 95% CI 0.75 to 5.48** vs aOR 0.77, 95% CI 0.24 to 2.47**			
					2.Normal for a woman to propose abstinence, 2nd follow-up vs. baseline	aOR 2.13, 95% CI 0.63 to 7.27** vs aOR 0.77, 95% CI 0.24 to 2.47**			
					3. A person can avoid HIV by abstaining from sex, 1st follow-up vs. baseline	aOR 3.52, 95% CI 1.20 to 10.28** vs aOR 1.11, 95% CI 0.43 to 2.86**			
					4. A person can avoid HIV by abstaining from sex, 2nd follow-up vs. baseline	aOR 5.80, 95% CI 1.58 to 21.27** vs aOR 1.11, 95% CI 0.43 to 2.86**			
					5. Received information from peer educators on how to use condoms at 1st follow-up vs. baseline	aOR 2.00, 95% CI 1.09 to 3.69** vs aOR 1.02, 95% CI 0.55 to 1.87**			
					6. Approve of individuals using condoms increased at 1st follow-up vs. baseline	aOR 2.04, 95% CI 1.28 to 3.26** vs aOR 1.04, 95% CI 0.66 to 1.65**			
					7. Normal for a woman to propose condom use	aOR 1.81, 95% CI 0.72 to 4.55** vs aOR 0.80,95% CI 0.33 to 1.91**			
					8. Intend to use condoms with regular partner increased at 1st follow-up vs. baseline	aOR 1.81, 95% CI 0.97 to 3.38** at 1st vs aOR 0.74, 95% CI 0.41 to 1.34**			
Mmbaga et al. [35]	*Structural* Harmful norms, lack of knowledge on SRH *Behavioral* Sexual risk behavior, early sexual initiation, IPV	Self-reported through questionnaire	*Theory* Social Cognitive Theory	Trained teachers, peer educators, healthcare providers	*Impact-based behavioral* Behavioral intervention vs. control groups at 12-months:				
					1. Plan to delay sex was similar among male adolescents			0.1497** vs. 0.1562	
					2. Condom use increased among boys based on GEE coefficient at 6 months			0.3209** vs. 0.1484	
Weiss et al. [36]	*Biological* Poor circumcision *Behavioral* Unsafe sexual practices, drug abuse	Self-reported through surveys, questionnaires and focus group discussions	*Model* Transtheoretical Model	VCT counselors and nurses	*Impact-based* Intervention vs. control:				
					1. VMMC coverage increased	aOR 2.45, 95% CI 1.24 to 4.90*			
					2. Experimental condition participants increased their condom use over time vs. no change for the control group	aOR 0.055,95% CI 0.01 to 0.10			
					3. No change in other risk behaviors, e.g., multiple partners, use of alcohol or drugs during sex				
Kaufman et al. (a) [38]	*Biological* Poor circumcision	*Circumcision* Clinic records *Intention to circumcise* self-reported questionnaire	*Theory* Social Learning Theory	Trained recently circumcised “coaches”, professional soccer players and community members	*Impact-based*				
					Found strong evidence of increased VMMC uptake for intervention compared to control group	OR 2.53, 95% CI 1.21 to 5.30**			
Gibbs et al. [39]	*Structural* Gender attitudes and unstable livelihoods	Self-reported through surveys, questionnaires and FGDs	*Model* Sustainable Livelihoods Framework	Peer facilitators	*Strength-based*				
					1. Men’s IPV reduced	aOR 0.71, 95% CI 0.51 to 0.97**			
					2. Reduced severe IPV	aOR 0.74, 95% CI 0.54 to 1.03*			
					3. No difference in men’s controlling behaviors			0.06, 95% CI − 0.51 to 0.63	
					4. No improvement in past month earnings			0.21, 95% CI − 0.42 to 0.83	
Kyegombe et al. [41]	*Structural* Harmful social norms promoting IPV *Behavioral* IPV, sexual risk behaviors	Self-reported through questionnaire	No theory/model mentioned	Not mentioned	*Impact-based* Men in intervention vs. control reported a broad range of HIV-protective behaviors:				
					1. Increased condom use adjusted relative risk. (models were adjusted for several socio-demographic factors, such as age and sex)		aRR 2.03, 95% CI 1.22 to 3.39**		
					2. Increased HIV testing		aRR 1.50, 95% CI 1.13 to 2.00**		
					3. Lower concurrent partners		aRR 0.60, 95% CI 0.37 to 0.97**		
					4. Increased joint decision-making		aRR 1.92, 95% CI 1.27 to 2.91**		
					5. Greater male participation in household tasks		aRR 1.48, 95% CI 1.09 to 2.01**		
Jemmott et al. [42]	*Structural* Norms that promote risky sex, poor condom-use skills and poor HIV/STI risk-reduction knowledge	Self-reported using a Likert scale	*Theory* Social Cognitive Theory	17 male IsiXhosa and English-speaking facilitators aged 25–53 years	*Impact-based* Participants reported higher odds of:				
					1. Consistent condom use	OR 1.32, 95% CI 1.03, 1.71*			
					2. Condom use at last vaginal intercourse	OR 1.40, 95% CI 1.08 to 1.82*			
Burnett et al. [46]	*Biological* Poor HIV/testing *Behavioral* Poor HIV prevention and treatment *Structural* HIV stigma and discrimination, harmful norms	Self-reported through questionnaire	*Theory* Self-efficacy Theory		*Outcome-based* Significant differences between pre- and post-intervention:				
					1. Overall HIV knowledge increased			↑from 0.08**, SE:0.02 vs. 0.45**, SE:0.07	
					2. Abstinence increased			↑from 0.14**, SE:0.04 vs − 0.69**, SE:0.07	
					3. Condom use increased			↑from 0.16**, SE:0.05 vs − 0.63**, SE:0.08	
					4. Ever had an HIV test increased	OR 10.96, 95% CI 4.59 to 26.15)			
					5. No change in knowing partner’s HIV status			0.06,SE:0.06 vs. 0.59, SE:0.08	
Kaufman et al. (b) [47]	*Behavioral* Cross-generational sex (CGS)	Self-reported through questionnaire	No theory/model mentioned	Trained facilitators	*Impact based behavioral*				
					No significant reduction in male involvement in intergenerational sexual relationships despite exposure to cross-generational sex campaigns: 1. 1–10 exposures in past 3 months compared to no exposure in past 3 months	OR 0.68, 95% CI 0.37 to 1.26			
					2. 11+ exposures in past 3 months	OR 1.11, 95% CI 0.61 to 2.01			

*aRR* adjusted relative risk, *aOR* adjusted odds, *SE* standard deviation

**p < 0.0001, *p < 0.05

### Description of Studies

Specific details of the 29 eligible studies are discussed in the summary below.

All interventions were from ten SSA countries i.e., South Africa [20–26, 34, 37, 39, 42, 43], Uganda [27, 30, 33, 41], Zimbabwe [38, 40, 48], Zambia [28, 36], Kenya [31], Tanzania [35, 47], Ethiopia [29], Nigeria [45], Eswatini [46] and Malawi [32]. One study was conducted in two countries [44], i.e., South Africa and Zambia. Of the 29 studies, 13 were randomized trials [22, 24, 34–42, 46, 48], six were quasi-experimental trials [28–30, 32, 43, 45], two were surveys [33, 47] and the remaining were either intervention evaluation studies or non-experimental studies, i.e., qualitative studies.

### Characteristics of Study Design

Theories and models help us to better understand the logic of an intervention. Eleven studies were guided by at least one theory to explain causal pathways of HIV risk, namely, social learning theory [20, 32, 38, 40], theory of positive normativity [20], social identity theory [21], theory of gender and power [29], theory of reasoned action [32], ecological systems theory [34], social cognitive theory [42], and theory of self-efficacy [46]. Models were also cited to explain or predict behaviors. Sixteen intervention studies utilized models to inform the intervention i.e., social ecology model [20], biomedical model of health [23, 27, 44], participatory learning model [24], human rights framework approach [25], Ubuntu model [26], health belief model [30], transtheoretical model [36], sustainable livelihoods model [39], gender-transformative approach [33, 41, 43] and the Sahwira (friendship) model [48]. Models simplify a theory or concept for a better understanding of the intervention. A combination of both theory and model was used in two studies [20, 46] to enhance their findings.

Intervention approaches were varied, with the majority of interventions using a combination-type approach. UNAIDS specifically defines combination HIV prevention as rights, evidence, and community-based programs that promote a combination of biomedical, behavioral, and structural interventions designed to meet the HIV prevention needs of specific people and communities [52]. The goal is to reduce the number of new HIV infections through coordinated activities with a greater sustained impact [53]. Messages include biomedical, norm-changing, behavioral, structural and psychosocial determinants of HIV transmission and vulnerability, all of which are strong predictors of health outcomes.

Intervention delivery was through mass media in seven intervention studies [20, 21, 29, 30, 33, 42, 47], community dialogue in 14 studies [20–26, 30, 33, 36, 39, 41, 42, 44], drama skits, poetry, puppetry and role play in ten studies [22, 27–29, 33, 35, 40, 42, 45, 46]. Eighteen studies were delivered through workshops and educational curricula [20, 22, 24–30, 32–36, 39, 40, 42, 46], and seven studies were delivered through interactive games [21, 22, 33, 37, 38, 40, 42].

Half of the interventions targeted a male population exclusively [20–23, 25–27, 29, 33, 36–38, 40, 42, 48], with two of these specifying heterosexual behaviors as their inclusion criteria [25, 33]. The remaining half targeted a mixed-gender audience, as specified in our study inclusion criteria. Eight interventions worked with trained facilitators [24, 26, 29, 30, 32, 35, 44, 47] and seven with peer counselors [25, 28, 33, 34, 39, 43, 48]. Two interventions had trained peer facilitators [28, 43]. Four interventions worked with trained medical personnel [23, 24, 27, 46], four with well-known soccer ambassadors to positively influence their population [20, 21, 38, 40], four with facilitators with desired characteristics such as religious leaders in The One Man Can Campaign [22], same-sex facilitators in the Stepping Stones intervention [24], facilitators who were not regular patrons of shebeens or drug addicts in the Champions League intervention [37], and recently circumcised coaches in Make the Cut + [38].

Interventions included both in- and out-of-school audiences. Twenty interventions [20–27, 29, 30, 32, 33, 36, 37, 39, 41, 42, 44, 47, 48] targeted out-of-school youth, and nine interventions targeted in-school youth [28, 31, 34, 35, 38, 40, 43, 45, 46]. Interventions were implemented in different contexts. Thirteen interventions were in urban areas [25, 28, 33–37, 39–41, 44, 45, 48], five in peri-urban areas [27, 29, 30, 38, 43], four in rural areas [22, 24, 26, 32], and five combined either urban, rural or peri-urban areas [20, 21, 23, 31, 47]. Two interventions did not specify their geographic areas [42, 46].

Overall, the exposure to the interventions varied with follow-up assessments ranging from as short as 2 weeks post-intervention in the Zambian Peer intervention study [28] to 5 years in the African Youth Alliance intervention study [30].

### Measured Outcomes

Reviewed studies investigated behavioral, norm-changing, biomedical and structural outcomes, reporting both positive and negative outcomes as well as no effect outcomes.

#### Behavioral Outcomes

Twenty-four intervention studies had measures relating to behavioral outcomes [20–22, 24–28, 30–37, 39–43, 45, 47, 48].

##### Condom Use

Of the interventions with a behavioral component, 15 studies measured outcomes related to condom use [22, 24–28, 30, 31, 34–36, 41, 42, 45, 48], with ten of these employing a randomized design [22, 24, 26, 34–36, 41, 42, 45, 48]. All findings were self-reported through questionnaires or surveys. A general improvement in condom use was reported in eight intervention studies [22, 24, 26, 27, 36, 41, 42, 45], with condom use being consistent in the Condom Technical Skill intervention study at 12-month follow-up [27]. Of the 15 study interventions, seven focused exclusively on males [22, 25–27, 36, 42, 48], and the remainder targeted a mixed-gender population. Condom use interventions had differential impacts by gender: condom use was higher in boys aged 12–14 years when compared to girls in the same age range in three mixed-gender intervention studies. For example, in the PATH-Kenyan Scouts intervention, the proportion of sexually active boys aged 15–18 years using condoms increased compared to girls of the same age [31]. Likewise, in the Africa Youth Alliance (AYA) intervention, condom use was higher in males than females at 5-year follow-up [30] as well as in the PREPARE intervention where condom use was higher in males and intention to use condoms was higher in females, at 6 and 12-month follow-up periods [35]. The impact was different depending on gender and school grade in the Healthwise intervention, a curriculum-based randomized trial for boys and girls (mean age 14) conducted in four urban secondary schools in South Africa [34]. Eighth grade lessons had a positive impact on girls, who responded better to the sexual risk and condom use self-efficacy lesson, compared to boys, whereas ninth-grade lessons had a positive impact on boys who self-reported higher condom use self-efficacy compared to girls at the 12-month follow-up. Improvements in intention to use condoms were recorded in the Zambian Peer Sexual Intervention study but this effect was not sustained during the 6 months that followed the intervention [28]. The Sahwira intervention reported no difference in self-reported condom use at 6-month follow-up when comparing pre- and post-intervention reports in both control and intervention conditions [48]. The intervention, a randomized controlled trial, targeted men 16 years and above and used the concept of ‘drinking partners’ to influence each other to avoid high-risk sexual encounters fueled by drinking at a beerhall.

##### Multiple Sex Partners

Multiple partner sex was measured in nine intervention studies [20, 22, 24, 27, 28, 41, 42, 45, 48], with five of these studies using a randomized design [22, 24, 41, 42, 48]. A self-reported decline in multiple sexual partnerships was reported in six studies at 12-month follow-up [20, 22, 24, 28, 41, 45]. Three interventions [27, 42, 48] did not have a significant effect on multiple sexual partnerships, with the Condom Technical Skills intervention reporting an increase in self-reported multiple sexual partners among ABYM at 6-month follow-up. No significant changes were noted over a 24-month period in mean number of sexual partners, 1.5 (1.3–1.7) vs. 1.5 (1.3–1.7), p = 0.98, and number of additional non-wife sex partners, 5.4 vs. 5.1, p = 0.98, compared to data prior to the intervention [48]. No significant gender differences were noted in these interventions.

##### Abstinence

Seven studies measured abstinence (both primary and secondary) with the aim of preventing early sexual debut [27, 28, 30, 32, 35, 43, 45]. Of these, four reported improved secondary abstinence [30, 35, 43, 45]. Although the African Youth Alliance (AYA) intervention reported improved secondary abstinence, the intervention had no effect in delaying sexual initiation among boys [30]. The Zambian Peer Intervention reported changes in beliefs about abstinence rather than in actual abstinence behavior among 14–23-year-old adolescent males and females at 9 month-follow-up [28]. Differential impacts by gender were noted in the Siyakha Nentsha intervention, with boys aged 14–16 years more likely to have remained sexually abstinent at the 18-month follow-up compared to girls of the same age [43]. The No Name intervention reported increased abstinence in both sexes at 3-year follow-up [45]. The PREPARE intervention influenced delaying self-reported sexual initiation among adolescent boys [35].

##### Intergenerational Sex

The Fataki intervention study measured intergenerational sex [47]. This intervention, which was the only one to measure intergenerational sex, noted no change in intergenerational sex for men exposed to a behavioral campaign more than ten times in the past 3 months (OR 1.11, 95% CI 0.61–2.01, p > 0.05) compared to the non-exposure group.

##### Alcohol and Drug Abuse

Four intervention studies measured alcohol and drug abuse targeting ABYM with a mean age of 16 years [21, 22, 24, 37]. Two of these were sports-based and reported a decline in substance abuse at 6 months post-intervention, measured using a rapid drug test before each soccer match [22, 37]. Alcohol and drug tests were randomly administered at the beginning of each soccer game. Games were held twice a week with competitive games on weekends. Reductions in alcohol consumption and alcohol-related behaviors were found in the One Man Can intervention [22], whereas less problem drinking was recorded in both sexes in the Stepping Stones intervention [24]. Findings were self-reported and follow-up periods ranged from 6 to 24 months.

##### Knowledge About HIV/AIDS

Knowledge about HIV and AIDS, including questions on prevention, risk factors and/or misconceptions, was assessed in five studies focusing on ABYM [24, 32, 40, 47, 48]. The average age of participants was 15 years. Improved HIV knowledge, attitudes, perceptions of social support, and HIV prevention knowledge were noted in three of these studies [24, 32, 40], with a differential gender impact in the Mzakhe ndi Mzakhe weekly educational sessions intervention [32], which found that the intervention had no effect on females aged 13–15 years compared to boys of the same age. Follow-up periods were from 6 months to 2 years. All outcomes were self-reported through surveys and group discussions.

#### Biomedical Outcomes

Biomedical interventions, which encompassed both clinical and medical outcomes, aimed to reduce HIV transmission. These included voluntary male circumcision (VMMC) and HIV counseling and testing.

##### VMMC

Four intervention studies focused on voluntary medical male circumcision, and all were effective [20, 23, 36, 38]. Results indicated that age was associated with VMMC in two of these studies, with younger participants more likely to undergo circumcision (aOR 2.45, 95% CI 1.24–4.90, p = 0.02) in the Spear and Shield intervention at both 6- and 12-month follow-up [36]. Exposure to education about VMMC was also associated with an increased likelihood of undergoing circumcision in this intervention. The Make the Cut + intervention reported increased VMMC uptake (OR 2.53, 95% CI 1.21–5.30, p = 0.01) compared to the control group [38]. Overall, 40% of participants in the experimental group underwent circumcision compared to 24% of control participants at 12-month follow-up. Outcomes were based on clinical records of circumcisions in both groups.

##### HIV Testing

Eight studies had outcomes related to HIV testing for ABYM [22, 24, 25, 32, 41, 44, 46, 48]. All of these studies found improvements in HIV testing uptake except the Sahwira intervention which reported no difference in HIV testing uptake pre- and post-intervention [48]. There was improved acceptance of testing as age increased in the PopART for youth study at 12 month-follow-up [44]. Testing was 29.2% before vs. 87.8% after the intervention in Zambia, and 21.3% vs. 77.3% in South Africa, respectively, with no measure of statistical significance provided. In another study, the SASA! intervention, intervention effects were greater in males than in females [41]. For men solely, HIV testing increased (aRR 1.50, 95% CI 1.13–2.00, p < 0.05) after the intervention compared to before. HIV testing outcomes were either self-reported or obtained from clinical records, with follow-up periods ranging from 12 months to 5 years for all studies.

#### Norm-Changing Outcomes

Eight studies evaluated norm-changing interventions [20, 22, 24, 25, 29, 31, 33, 39], which sought to change attitudes and social beliefs about HIV/AIDS risk. Social environments and social norms that heighten risky sexual behaviors place young men and women at extremely high risk of HIV/STI acquisition and onward transmission. These include stereotypical norms of masculinity, such as multiple sexual partnerships and condoning physical and sexual violence to dominate partners in relationships. Gender norms deter men from accessing health services, resulting in poor uptake of preventive care services, including HIV testing. Of note, males (18–35 years) embraced attitudes for equality with women and perceived their male identity differently in ways that reduce violence against women and intimate partners in three interventions [20, 22, 25]. The interventions had no effect in two studies [25, 39]. There was a marked increase and decrease post-intervention in the proportion of men who believed that men should make final household decisions (50% vs. 38%, p = 0.036) and that women who carry condoms are easy (36% vs. 20%, p = 0.004) compared to pre-intervention, respectively, in the Men as Partners intervention campaign [25]. The combined Stepping Stones and Creating Futures intervention found no evidence of change in men’s controlling behaviors toward women (β = 0.06, 95% CI − 0.51 to 0.63, p = 0.839) [39]. This intervention also found lower odds of interpersonal violence (IPV) post-compared to pre-intervention (aOR 0.71, 95% CI 0.51–0.97, p < 0.001) [39]. Outcomes were measured by means of the Gender-Equitable Men (GEM) Scale in three interventions [22, 29, 31] and validated through police or hospital records of physical or sexual violence committed during the preceding 6 months in three interventions [20, 22, 24] or self-reported in two interventions [25, 39]. Follow-up periods for these studies ranged from 6 months to 3 years.

#### Livelihood-Strengthening Outcomes

Young people, not formally employed or educated, face exceedingly high levels of IPV and an increased likelihood of engaging in risky social interactions, raising the potential for HIV acquisition. Interventions designed to reduce vulnerability through enhancing livelihoods and financial independence as well as offering social protection have been shown to reduce HIV vulnerability and IPV [54].

Only two interventions had a social protection component, and both reported improved outcomes compared to pre-intervention periods [39, 43]. The Stepping-Stones and creating futures intervention found no improvement in past month earnings savings for both men and women at 24-month follow-up (β = 0.21, 95% CI − 0.42 to 0.83, p = 0.521) [39]. The Siyakha Nenthsa Programme recorded improved budgeting and planning skills in school-going boys and girls (14–16 years) only in the intervention group at 18-month follow-up [43]. Participants were more likely to have attempted to open a bank account when compared to the control group. Men’s self-esteem improved and criminal behaviors were reduced because men were self-sufficient due to the Stepping Stones and Creating Futures intervention [39]. Men reported feeling less shame about lack of work and less stealing in the past week due to hunger.

## Discussion

This scoping review examined 29 studies that evaluated sexual risk-reduction interventions in ABYM between the ages of 10 and 24 in nine SSA countries. Our results show that behavioral interventions were moderately successful in improving condom use [22, 24, 26, 27, 36, 41, 42, 45]. However, the review also showed that sexual risk-reduction interventions (especially those including condom promotion) could encourage multiple sexual partnerships, thereby increasing HIV transmission risk. For example, the Condom Technical Skills intervention improved condom use, while simultaneously increasing multiple partner sexual activity among participants [27]. This spike in multiple partnership sexual activity may have been brought about by an over-reliance on condom safety, while neglecting related aspects of sexual behavior (frequency of sex, number of sex partners—multiple and concurrent). The more partners one has, the greater the odds of acquiring HIV. Condoms may help in ensuring safety, but this is only applicable when they are used correctly and consistently.

This review found that interventions had a positive effect on the reduction of multiple sex partners [20, 22, 24, 28, 41, 45]. However, in the interventions included in this review, sustaining such a positive outcome was a challenge. One study showed an increase in multiple sex partners post-intervention, possibly indicating neglect in tackling normative drivers that underpin sexual risk practices [27].

In addition, the review highlighted a differential impact across gender. The Healthwise program found that boys had greater condom use self-efficacy than girls both at baseline and throughout the study [34]. Differences may be because 8th-grade lessons taught skills such as decision-making and negotiation which might appeal more to girls, whereas 9th-grade lessons focused on condom use within sexual relationships which might be more salient to boys than girls. However, the African Youth Alliance intervention substantially increased condom use, consistency of condom use, and contraceptive use among female but not male participants [30].

Although the above results are gender-specific, mixed-gender intervention approaches should be adopted. Their strength lies in their ability to enable ABYM to engage with AGYW to explore and reframe gender and sex roles, assumptions, and decision-making in a safe, structured setting [39]. Findings also suggest the importance of introducing topics in sex-segregated groups because men and women in the early phases of sex most likely engage with different levels of sexual awareness; thus, gender-specific interventions may be appropriate, at least initially. For instance, girls often report earlier sexual debut than boys [55]; therefore, program goals for men and women should not overlap at different developmental stages [56]. Welbourn also favors such an approach, pointing to the usefulness of engaging existing culturally defined community groups, generally divided along gender and age lines, which allows each group to have safe private time and space to explore their own concerns [57].

Male-specific positive outcomes emphasized the potential of brotherhood bonds to initiate change which opened up spaces to discuss men’s harmful sexual practices while trying to restore a sense of dignity among men [23]. Through such interactive and communicative group spaces, participants constructed messages and meanings of their social realities, with the potential for transformation toward healthy masculine attitudes. As noted by Figueroa and colleagues, the ideal model of development communication is one “based on dialogue versus monologue, horizontal versus vertical information sharing, equitable participation, local ownership, empowerment, and social versus individual change” [23]. In other words, interventions should be tailored to the target population with their participation and input to optimize their success.

Interventions are likely to be most effective when they are age-appropriate and tailored to the cognitive level of the adolescent. More specifically, Spear and Shield found that younger participants were more likely to undergo circumcision compared to their older peers [36]. However, in the Make the Cut + intervention, there was evidence of increased uptake of VMCC with age in both study arms [38]. Even though the above findings seem contradictory, other research indicates that sexual risk-reduction interventions that generally target pre- or early adolescence, irrespective of the focal outcome, produce more positive outcomes than those targeting late adolescence [58]. Targeting pre- or early adolescence for sexual risk-reduction interventions is also more feasible because a significant proportion of young people are less likely to have initiated sex at this stage [58].

Interventions included in the review have also shown to be effective in improving attitudes and norms [20, 22, 25, 39]. Norms have long been recognized as critical barriers to HIV prevention behaviors. Addressing gender inequalities may reduce young men’s perpetration of gender and sexual violence and encourage young men to engage in protected sex [25]. Combined with livelihood-strengthening outcomes, intervention effects may be bolstered. For example, the It’s Our Futures Too intervention (a combination intervention approach) noted significant and positive differences in financial independence, overall HIV knowledge, self-efficacy related to abstinence and condom use, and knowing one’s own HIV status between the intervention and control groups [46]. These findings support recent evidence from Uganda suggesting that a combination social protection response may be more effective than unidimensional programming [59, 60]. A successful intervention is more likely to have compounding and synergistic effects in reducing HIV transmission below the reproductive rate necessary to achieve HIV epidemic control [61–63].

Interventions that entail the use of friendship networks show promise in influencing peer norms and behavior among ABYM both in- and out-of-school. Boys create and seek out spaces among their male peers from which to cultivate their masculinities through heterosexual discourses, including being ‘at risk’ of getting HIV [64]. The success of Stepping Stones was clearly associated with peer-to-peer education aimed at gender transformational change and HIV risk-reduction behaviors, particularly among men, when offered viable alternative normative behaviors [24]. The Stepping Stones and creating futures study showed reductions in IPV-see Gibbs et al. [34]. The Brother’s for Life intervention also reached men in their interactive group/friendship networks and showed some positive trends toward reducing rape and domestic violence through enforcing positive norms and reducing concurrent sexual partnerships [20].

More generally, a peer network approach emphasizes the potential of brotherhood bonds to initiate change, opening spaces to discuss men’s relations with their intimate partners, while trying to restore their sense of dignity [23]. Through such interactive and communicative group spaces, ABYM can construct messages and meanings of their social realities, with the potential for transformation to adopting healthy attitudes and behaviors.

Outcomes from this review suggest that although communication channels, such as the media (TV, newsprint, billboards), can perpetuate harmful gender norms, they also can be potential avenues for shifting attitudes, norms and behaviors. The Brothers for Life intervention [20] and Grassroots intervention [40] used highly innovative, action-oriented group-based activities such as warm-up games, role-plays, radio and TV discussions, and brainstorming. These interventions reported improvements in HIV prevention knowledge and correct and consistent condom use.

Although structural interventions are often critiqued as ‘social development’ rather than focused health interventions [65], this review showed how such interventions can alter the context of young people’s HIV risk through improving personal agency. The Siyakha Nentsha intervention found increased autonomy around financial decision-making [43]. Boys in the intervention group were more likely to have remained sexually abstinent between survey rounds, and those who did have sex reported fewer sexual partners than boys in the control group.

Use of facilitators who are well-regarded to disseminate messages contributed to intervention success. In the Learning Centre Initiative, religious leaders contributed to the intervention’s success, noteworthy given that religion could also be an impediment to sexual health promotion [33]. The Brothers for Life Campaign used soccer celebrities and coaches who were perceived as displaying upstanding behavior as trusted trendsetters whose actions, attitudes, and views influenced their peers [20]. In the Champions League intervention, use of same-sex facilitators as well as peer counselors proved beneficial as participants felt comfortable discussing personal issues with members of the same sex [37].

Program sustainability, “the ability to maintain programing and its benefits over time” [66] beyond the intervention period was an issue in the studies reviewed. Many interventions could not be sustained after implementation. For example, the Zambian Peer Intervention found changes in normative beliefs about abstinence, but these changes could not be sustained 6 months after the intervention. Maintaining effective programs and practices is critical for achieving health benefits for the intended population in which positive change is desired [36].

### Strengths and Limitations of the Scoping Review and Studies Reviewed

The strength of this review is that it covered a period of 20 years of published studies focusing on ABYM populations in- and out-of-school in SSA. The review focused on studies using a variety of methodologies in different settings and identified interventions that showed both positive and negative outcomes (reducing sexual risk behaviors and associated attitudes and norms) using different measures. Additional studies that included young males might have been missed in our search for several reasons. We excluded interventions from the review if study results were not stratified by age and/or sex. Some studies that included age and sex in their analysis were ultimately excluded for failing to specifically report intervention outcome effects in young adult males. Analysis of intervention findings by age and gender sub-groups is critical to better reflect the diversity in risk behaviors among ABYM.

A key weakness of many studies in the review is the reliance on self-reported measures of behavior change. Self-reported outcomes reduce confidence in demonstrating intervention efficacy. When self-reported data and biomarkers are readily available and easy to collect, a combination of these measures is recommended as a reliable representative of sexual risk behavior [67].

There were few commonalities in the study designs of interventions in this review (type and content), perhaps suggesting little consensus on the optimal approach to evaluating these interventions. Out of the 29 studies included in the review, only 14 were randomized trials. Future interventions need to adopt a more rigorous methodology in the design and measurement of risk behaviors. Using outcome measures that are standard across trials may permit a more uniform comparison of interventions.

Interventions in this review included both in- and out-of-school interventions. Schools are an ideal place to reach ABYM because this is where they are likely to receive appropriate sexuality information and education. Although in-school interventions may be feasible and cost-effective, out-of-school interventions are equally important because this ABYM population may be more vulnerable and exposed to sexual risk-taking behavior. Out-of-school interventions can target ABYM through informal community programs, e.g., street theater and music. Interventions such as Sonke Gender Justice’s One-Man Can and Engender Health’s Men as Partners (MAP) targeted ABYM through informal community programs [22, 25].

Studies included in the review highlighted the paucity of structural interventions, specifically those with an economic link such as cash transfers. Only the Stepping Stones and Creating Futures intervention had an economic element [39]. The remaining were norm-based interventions. As such, there is need for interventions to adopt structural approaches that can alter the context of young people’s HIV acquisition through economic empowerment initiatives as well as those that change the social and political contexts that influence the drivers or mediators of HIV.

Several studies based their interventions on behavioral theories and models, e.g., the Health Belief Model [30], Social Learning [20, 32, 38, 40] and Theory of Reasoned Action [32]. These theories have been criticized for being simplistic and reductionist in their analysis of sexual behavior [12, 13] as they do not consider the contextual and structural determinants of sexual behavior. Working with reductionist theoretical frameworks can lead to limited effective intervention outcomes. Developing more explicit links between theories of HIV risk and possible intervention pathways toward behavior change in different HIV contexts and populations would be an important step for future research.

Sustained behavior change also remains a challenge in the interventions reviewed. There is a need to identify and describe existing facilitators or barriers to outcomes to better understand implementation processes, promote the use of impactful interventions, and advance the field of dissemination and implementation science. Lack of appropriate conceptualization of interventions from the outset compromises program sustainability.

## Conclusion

This review indicates that sexual-risk interventions engaging ABYM in SSA show some promise and identifies ways to build and strengthen good practices, particularly those that encourage combination-type sexual risk-reduction interventions (biological, behavioral and structural components). The review found a positive impact of interventions on condom use, reduction of multiple sexual partners, abstinence, alcohol and drug abuse, and HIV testing as well as livelihood-strengthening interventions. Where gender, age and grade variables were employed in study designs, effects were mixed. Future studies need to pay more attention to these variables when designing and evaluating interventions. However, where interventions showed positive evidence of reducing risky sexual behaviors, change was not sustained. Future interventions require more longitudinal studies (including a qualitative and process-level methodology) to document why health behaviors may not be sustained in specific contexts post-intervention. Engaging ABYM in HIV prevention interventions should occur in ways that do no harm, but promote gender and sexual diversity, equality, and health for all.

## Data Availability

All data relevant to the study are included in the article or uploaded as supplementary information. Any further data required are available on request from the corresponding author.

## References

[1] Govender K, Masebo WGB, Nyamaruze P, Cowden RG, Schunter BT, Bains A (2018). HIV prevention in adolescents and young people in the Eastern and southern African region: a review of key challenges impeding actions for an effective response. Open AIDS J.

[2] Melesse DY, Cane RM, Mangombe A, Ijadunola MY, Manu A, Bamgboye E (2021). Inequalities in early marriage, childbearing and sexual debut among adolescents in sub-Saharan Africa. Reprod Health.

[3] UNAIDS. Country fact sheet. 2017 [cited 2019 Feb 08]. Available from: file:///C:/Users/210551048/Downloads/Country%20factsheets%20Eswatini%202017.pdf.

[4] UNAIDS. Miles to go: the response to HIV in Eastern and Southern Africa. 2018 [cited 2019 Jan 23]. Available from: www.unaids.org/sites/default/files/media_asset/miles-to-go_eastern-and-southern-africa_en.pdf.

[5] Gafos M, Beattie T, Stoebenau K, Baron D, Weiner R, Wamoyi J, Govender K, Poku N (2021). Addressing structural drivers of HIV among young people in Eastern and Southern Africa: evidence, challenges and recommendations for advancing the field. Preventing HIV among young people in Eastern and Southern Africa: emerging evidence and intervention strategies.

[6] Gittings L, Hodes R, Colvin CJ, Zungu N, Govender K, Poku N (2021). Things less spoken—HIV research with adolescent boys and young men: Implications for theory, policy and practice. Preventing HIV among young people in Eastern and Southern Africa: emerging evidence and intervention strategies.

[7] Mantell JE, Hoffman S, Low S, Kelvin E, Govender K, Poku N (2021). Are adolescent boys and young men being left behind? Missing discourse and missed opportunities for engagement in HIV prevention in Eastern and Southern Africa. Preventing HIV among young people in Eastern and Southern Africa: emerging evidence and intervention strategies.

[8] Michielsen K, Chersich MF, Luchters S, De Koker P, Van Rossem R, Temmerman M (2010). Effectiveness of HIV prevention for youth in sub-Saharan Africa: systematic review and meta-analysis of randomized and nonrandomized trials. AIDS.

[9] Cowan FM, Pascoe SJS, Langhaug LF, Mavhu W, Chidiya S, Jaffar S (2010). The Regai Dzive Shiri project: results of a randomized trial of an HIV prevention intervention for youth. AIDS.

[10] Dennison S, Leclerc B (2011). Developmental factors in adolescent child sexual offenders: a comparison of nonrepeat and repeat sexual offenders. Crim Justice Behav.

[11] Grant M, Booth A (2009). A typology of reviews: an analysis of 14 review types and associated methodologies. Health Inf Lib J.

[12] Mwale M, Muula AS (2017). Systematic review: a review of adolescent behavior change interventions [BCI] and their effectiveness in HIV and AIDS prevention in sub-Saharan Africa. BMC Public Health.

[13] Gallant M, Maticka-Tyndale E (2004). School-based HIV prevention programmes for African youth. Soc Sci Med.

[14] Govender K, Beckett S, Reddy T, Cowden RG, Cawood C, Khanyile D (2022). Association of HIV intervention uptake with HIV prevalence in adolescent girls and young women in South Africa. JAMA Netw Open.

[15] UNAIDSb. HIV prevention among adolescent girls and young women. 2016 [cited 2018 May 23]. Available from: www.unaids.org/sites/default/files/media_asset/UNAIDS_HIV_prevention_among_adolescent_girls_and_young_women.pdf.

[16] Arksey H, O’Malley L (2005). Scoping studies: towards a methodological framework. Int J Soc Res Methodol.

[17] Peters MDJ, Marnie C, Tricco AC, Pollock D, Munn Z, Alexander L (2020). Updated methodological guidance for the conduct of scoping reviews. JBI Evid Synth.

[18] Souto RQ, Khanassov V, Hong QN, Bush PL, Vedel I, Pluye P (2015). Systematic mixed studies reviews: updating results on the reliability and efficiency of the Mixed Methods Appraisal Tool. Int J Nurs Stud.

[19] Hong QN, Fàbregues S, Bartlett G, Boardman F, Cargo M, Dagenais P (2018). The Mixed Methods Appraisal Tool (MMAT) version 2018 for information professionals and researchers. Educ Inf.

[20] Myers L, Hajiyiannis H, Clarfelt A, Bessenaar T, Motuba T, Mashale R (2012). Audience reception analysis of the national Brothers for Life mass media campaign.

[21] Collinge J, Delate R, Figueroa M (2013). Talking man to man, the story of Brothers for Life.

[22] Pettifor A, Lippman SA, Selin AM, Peacock D, Gottert A, Maman S (2015). A cluster randomized-controlled trial of a community mobilization intervention to change gender norms and reduce HIV risk in rural South Africa: study design and intervention. BMC Public Health.

[23] Figueroa ME, Kincaid DL (2013). The impact of the Brothers for Life Campaign on male circumcision intention in South Africa.

[24] Jewkes R, Nduna M, Levin J, Jama N, Dunkle K, Khuzwayo N (2006). A cluster randomized-controlled trial to determine the effectiveness of Stepping Stones in preventing HIV infections and promoting safer sexual behaviour amongst youth in the rural Eastern Cape, South Africa: trial design, methods and baseline findings. Trop Med Int Health.

[25] Ditlopo P, Mullick S, Askew I, Vernon R, Sibeko S. Testing the effectiveness of the Men as Partners Program (MAP) in Soweto, South Africa. *FRONTIERS* Final Report. 2007. p. 23–38.

[26] York M (2014). Transforming masculinities: a qualitative study of a transformative education programme for young Zulu men and boys in rural KwaZulu-Natal. J Pan Afr Stud.

[27] Kajubi P, Kamya MR, Kamya S, Chen S, McFarland W, Hearst N (2005). Increasing condom use without reducing HIV risk: results of a controlled community trial in Uganda. J Acquir Immune Defic Syndr.

[28] Agha S, Vanrossem R (2004). Impact of a school-based peer sexual health intervention on normative beliefs, risk perceptions, and sexual behavior of Zambian adolescents. J Adolesc Health.

[29] Pulerwitz J, Hughes L, Mehta M, Kidanu A, Verani F, Tewolde S (2015). Changing gender norms and reducing intimate partner violence: results from a quasi-experimental intervention study with young men in Ethiopia. Am J Public Health.

[30] Karim AM, Williams T, Patykewich L, Ali D, Colvin CE, Posner J (2009). The impact of the African Youth Alliance program on the sexual behavior of young people in Uganda. Stud Fam Plan.

[31] PATH. Evaluation summary: changing gender norms of Kenyan scouts. PATH; 2012.

[32] Dancy BL, Jere DL, Kachingwe SI, Kaponda CPN, Norr JL, Norr KF (2014). HIV risk reduction intervention for rural adolescents in Malawi. J HIV AIDS Soc Serv.

[33] Stern E, Pascoe L, Shand T, Richmond S (2015). Lessons learned from engaging men in sexual and reproductive health as clients, partners and advocates of change in the Hoima district of Uganda. Cult Health Sex.

[34] Coffman DL, Smith EA, Flisher AJ, Caldwell LL (2011). Effects of HealthWise South Africa on condom use self-efficacy. Prev Sci.

[35] Mmbaga E, Kajula L, Aarø L, Kilonzo M, Wubs A, Eggers S (2017). Effect of the PREPARE intervention on sexual initiation and condom use among adolescents aged 12–14: a cluster randomised controlled trial in Dar es Salaam, Tanzania. BMC Public Health.

[36] Weiss S, Zulu R, Jones D, Redding C, Cook R, Chitalu N (2015). The Spear and Shield intervention to increase the availability and acceptability of voluntary medical male circumcision in Zambia: a cluster randomized controlled trial. Lancet HIV.

[37] Rotheram-Borus MJ, Tomlinson M, Durkin A, Baird K, DeCelles J, Swendeman D (2016). Feasibility of using soccer and job training to prevent drug abuse and HIV. AIDS Behav.

[38] Kaufman ZA, DeCelles J, Bhauti K, Hershow RB, Weiss HA, Chaibva C (2016). A sport-based intervention to increase uptake of voluntary medical male circumcision among adolescent male students: results from the MCUTS 2 cluster-randomized trial in Bulawayo, Zimbabwe. J Acquir Immune Defic Syndr.

[39] Gibbs A, Washington L, Abdelatif N, Chirwa E, Willan S, Shai N (2020). Stepping Stones and Creating Futures intervention to prevent intimate partner violence among young people: cluster randomized controlled trial. J Adolesc Health.

[40] Botcheva L, Huffman L. Grassroot Soccer Foundation. HIV/AIDS Education Program: an intervention in Zimbabwe. The children’s health council outcomes research consulting service. 2004. p. 4–35.

[41] Kyegombe N, Abramsky T, Devries KM, Starmann E, Michau L, Nakuti J (2014). The impact of SASA! A community mobilization intervention, on reported HIV-related risk behaviors and relationship dynamics in Kampala, Uganda. J Int AIDS Soc.

[42] Jemmott JB, Jemmott LS, O’Leary A, Ngwane Z, Icard LD, Heeren GA (2014). Cluster-randomized controlled trial of an HIV/sexually transmitted infection risk-reduction intervention for South African men. Am J Public Health.

[43] Hallman K, Roca E. Siyakha Nentsha: building economic, health, and social capabilities among highly vulnerable adolescents in KwaZulu-Natal, South Africa. Population Council. 2011. p. 1–4.

[44] Shanaube K, Macleod D, Chaila MJ, Mackworth-Young C, Hoddinott G, Schaap A (2021). HIV care cascade among adolescents in a “test and treat” community-based intervention: HPTN 071 (PopART) for Youth study. J Adolesc Health.

[45] Aderibigbe S, Araoye M (2008). Effect of health education on sexual behaviour of students of public secondary schools in Ilorin, Nigeria. Eur J Sci Res.

[46] Burnett SM, Weaver MR, Mody-Pan PN, Thomas LAR, Mar CM (2011). Evaluation of an intervention to increase human immunodeficiency virus testing among youth in Manzini, Swaziland: a randomized control trial. J Adolesc Health.

[47] Kaufman MR, Mooney A, Kamala B, Modarres N, Karam R, Ng’wanansabi D (2013). Effects of the Fataki campaign: addressing cross-generational sex in Tanzania by mobilizing communities to intervene. AIDS Behav..

[48] Fritz K, McFarland W, Wyrod R, Chasakara C, Makumbe K, Chirowodza A (2011). Evaluation of a peer network-based sexual risk reduction intervention for men in beer halls in Zimbabwe: results from a randomized controlled trial. AIDS Behav.

[49] Munn Z, Tufanaru C, Aromataris E (2014). JBI’s systematic reviews: data extraction and synthesis. Am J Nurs.

[50] Jordan Z, Lockwood C, Munn Z, Aromataris E (2019). The updated Joanna Briggs Institute Model of Evidence-Based Healthcare. Int J Evid Based Healthc.

[51] dos Santos WM, Secoli SR, de Araújo Püschel VA (2018). The Joanna Briggs Institute approach for systematic reviews. Rev Lat Am Enfermagem..

[52] UNAIDS. State of the AIDS epidemic. UNAIDS; 2009.

[53] Avert. HIV and AIDS in South Africa. AVERT; 2019. Available from: https://www.avert.org/professionals/hiv-around-world/sub-Saharan-africa/south-africa. Accessed 1 Aug 2019.

[54] Cluver LD, Orkin FM, Meinck F, Boyes ME, Yakubovich AR, Sherr L (2016). Can social protection improve sustainable development goals for adolescent health?. PLoS ONE..

[55] Wamoyi J, Mshana G, Mongi A, Neke N, Kapiga S, Changalucha J (2014). A review of interventions addressing structural drivers of adolescents’ sexual and reproductive health vulnerability in sub-Saharan Africa: implications for sexual health programming. Reprod Health.

[56] Orchowski LM, Edwards KM, Hollander JA, Banyard VL, Senn CY, Gidycz CA (2020). Integrating sexual assault resistance, bystander, and men’s social norms strategies to prevent sexual violence on college campuses: a call to action. Trauma Violence Abuse.

[57] Welbourn A. Gender, sex and HIV: how to address issues that no-one wants to hear about. In: Tant qu’on a la santé. Graduate Institute Publications; 2016. p. 195–227.

[58] Mwale M, Muula AS (2017). Systematic review: a review of adolescent behavior change interventions [BCI] and their effectiveness in HIV and AIDS prevention in sub-Saharan Africa. BMC Public Health.

[59] Ssewamala FM, Han C-K, Neilands TB, Ismayilova L, Sperber E (2010). Effect of economic assets on sexual risk-taking intentions among orphaned adolescents in Uganda. Am J Public Health.

[60] Ssewamala FM, Nabunya P, Mukasa NM, Ilic V, Nattabi J (2014). Integrating a mentorship component in programming for care and support of AIDS-orphaned and vulnerable children: lessons from the Suubi and Bridges Programs in sub-Saharan Africa. Glob Soc Welf.

[61] Piot P, Bartos M, Larson H, Zewdie D, Mane P (2008). Coming to terms with complexity: a call to action for HIV prevention. Lancet.

[62] Katsidzira L, Hakim JG (2011). HIV prevention in southern Africa: why we must reassess our strategies? HIV prevention in southern Africa. Trop Med Int Health.

[63] UNAIDS. AIDSinfo. 2020 [cited 2019 Jan 20]. Available from: http://aidsinfo.unaids.org/.

[64] Govender K (2011). The cool, the bad, the ugly, and the powerful: identity struggles in schoolboy peer culture. Cult Health Sex.

[65] Gupta GR, Parkhurst JO, Ogden JA, Aggleton P, Mahal A (2008). Structural approaches to HIV prevention. Lancet.

[66] Walugembe DR, Sibbald S, Le Ber MJ, Kothari A (2019). Sustainability of public health interventions: where are the gaps?. Health Res Policy Syst.

[67] Corno L (2019). Risky sexual behaviors: biological markers and self-reported data. Economica.

